# Effects of Recent Prior Dengue Infection on Risk and Severity of Subsequent SARS-CoV-2 Infection: A Retrospective Cohort Study

**DOI:** 10.1093/ofid/ofae397

**Published:** 2024-07-13

**Authors:** Nicole Tang, Jue Tao Lim, Borame Dickens, Calvin Chiew, Lee Ching Ng, Po Ying Chia, Yee Sin Leo, David Chien Lye, Kelvin Bryan Tan, Liang En Wee

**Affiliations:** National Centre for Infectious Diseases, Singapore, Singapore; Yong Loo Lin School of Medicine, National University of Singapore, Singapore, Singapore; National Centre for Infectious Diseases, Singapore, Singapore; Lee Kong Chian School of Medicine, Nanyang Technological University, Singapore, Singapore; Saw Swee Hock School of Public Health, National University of Singapore, Singapore, Singapore; National Centre for Infectious Diseases, Singapore, Singapore; Ministry of Health, Singapore, Singapore; Saw Swee Hock School of Public Health, National University of Singapore, Singapore, Singapore; Environmental Health Institute, National Environment Agency, Singapore, Singapore; National Centre for Infectious Diseases, Singapore, Singapore; Lee Kong Chian School of Medicine, Nanyang Technological University, Singapore, Singapore; Department of Infectious Diseases, Tan Tock Seng Hospital, Singapore, Singapore; National Centre for Infectious Diseases, Singapore, Singapore; Yong Loo Lin School of Medicine, National University of Singapore, Singapore, Singapore; Lee Kong Chian School of Medicine, Nanyang Technological University, Singapore, Singapore; Saw Swee Hock School of Public Health, National University of Singapore, Singapore, Singapore; Department of Infectious Diseases, Tan Tock Seng Hospital, Singapore, Singapore; National Centre for Infectious Diseases, Singapore, Singapore; Yong Loo Lin School of Medicine, National University of Singapore, Singapore, Singapore; Lee Kong Chian School of Medicine, Nanyang Technological University, Singapore, Singapore; Department of Infectious Diseases, Tan Tock Seng Hospital, Singapore, Singapore; National Centre for Infectious Diseases, Singapore, Singapore; Lee Kong Chian School of Medicine, Nanyang Technological University, Singapore, Singapore; Saw Swee Hock School of Public Health, National University of Singapore, Singapore, Singapore; Ministry of Health, Singapore, Singapore; Duke-NUS Graduate Medical School, National University of Singapore, Singapore, Singapore; National Centre for Infectious Diseases, Singapore, Singapore; Duke-NUS Graduate Medical School, National University of Singapore, Singapore, Singapore; Department of Infectious Diseases, Singapore General Hospital, Singapore, Singapore

**Keywords:** COVID-19, dengue, Omicron, SARS-CoV-2, vaccination

## Abstract

**Background and Aims:**

Elucidating whether prior dengue potentially confers cross-protection against COVID-19 is of public health importance in tropical countries at risk of overlapping dengue and COVID-19 epidemics. However, studies to date have yielded conflicting results. We aimed to assess effects of recent prior dengue infection on risk and severity of subsequent SARS-CoV-2 infection among adult Singaporeans.

**Methods:**

A retrospective cohort study including all adult Singaporeans aged ≥18 years was conducted from 1 July 2021 through 31 October 2022, when a dengue outbreak driven by the DENV3 serotype preceded subsequent waves of SARS-CoV-2 Delta/Omicron transmission in Singapore. SARS-CoV-2 and dengue infection status were classified using national registries. Cox regression models adjusted for demographics, COVID-19 vaccination status, comorbidity, and socioeconomic-status were used to assess risks and severity (hospitalization, severe illness) of SARS-CoV-2 infection occurring after previous recorded dengue infection.

**Results:**

A total of 3 366 399 individuals were included, contributing 1 399 696 530 person-days of observation. A total of 13 434 dengue infections and 1 253 520 subsequent SARS-CoV-2 infections were recorded; with an average of 94.7 days (standard deviation = 83.8) between dengue infection and SARS-CoV-2 infection. Preceding dengue infection was associated with a modest increase in risk of subsequent SARS-CoV-2 infection (adjusted hazards ratio [aHR] = 1.13; 95% confidence interval [CI], 1.08–1.17), and significantly elevated risk of subsequent COVID-19 hospitalization (aHR = 3.25; 95% CI, 2.78–3.82) and severe COVID-19 (aHR = 3.39; 95% CI, 2.29–5.03**)**.

**Conclusions:**

Increased risk of SARS-CoV-2 infection and adverse COVID-19 outcomes were observed following preceding dengue infection in a national population-based cohort of adult Singaporeans. This observation is of significance in tropical countries with overlapping dengue and COVID-19 outbreaks.

## Teaser

In a national population-based cohort of adult Singaporeans, during overlapping dengue and COVID-19 outbreaks driven by concurrent emergence of DENV3 and SARS-CoV-2 Delta/Omicron variants, increased risk of SARS-CoV-2 infection, hospitalization, and severe COVID-19 was observed following recent preceding dengue infection.

As COVID-19 moves toward endemicity, surges in SARS-CoV-2 transmission can potentially overlap with seasonal epidemics of other infectious diseases, further increasing the burden on health care systems, particularly in less well-resourced low-and-middle-income countries. Dengue is an example; dengue epidemics occur seasonally in tropical regions where dengue is endemic and overlapping outbreaks of dengue and COVID-19 are well-described [1, 2]. Double epidemics of dengue and COVID-19 were observed across multiple countries in tropical and subtropical Asia [1, 2], resulting in significant strain on hospitals and health care systems. Several studies at the onset of the COVID-19 pandemic raised the possibility of synergistic interaction between dengue and COVID-19 epidemics [3, 4]; however, discordant results have been reported. In 2 separate cohorts from Amazonian Brazil recruited during transmission of the ancestral SARS-CoV-2 B.1.1.33 variant, whereas serological proof of prior dengue infection was associated with twice the risk of clinically apparent COVID-19 in a cohort [3], dengue was observed to confer a protective effect against severe COVID-19 in a separate cohort, with individuals with prior self-reported dengue having lower risk of COVID-19 mortality (adjusted hazard-ratio [aHR] = 0.44) [4].

Several reasons may account for these differences. Misclassification may arise in the absence of diagnostic confirmation for both dengue and SARS-CoV-2 infection, given limited access to laboratory testing, particularly in the case of mildly symptomatic individuals in underresourced settings. Additionally, although antigenic cross-reactivity between dengue and SARS-CoV-2 raises the possibility of either increased risk of severe illness through antibody-dependent-like enhancement after sequential infection or decreased risk arising from cross-protective immunity, there is significant heterogeneity reported in the literature, with varying estimates of antibody cross-reactivity between dengue and SARS-CoV-2 and the extent of neutralizing ability conferred by such antibodies [5–8]. The majority of these studies were also conducted during transmission of ancestral SARS-CoV-2 strains [5–8]; however, subsequent emergence of the SARS-CoV-2 Omicron variant has since resulted in significant antigenic shift [9]. Cohort studies evaluating the impact of prior dengue infection on severity of subsequent SARS-CoV-2 infection were conducted before the rollout of COVID-19 vaccination [3, 4]; as such, results may not be fully generalizable to the current era of widespread vaccine availability. Given the burden of overlapping SARS-CoV-2 and dengue transmission in tropical countries during COVID-19 endemicity, elucidating the bidirectional impact of protection in COVID-19 and dengue is of public health importance. Using national registry data for test-confirmed dengue and SARS-CoV-2 infections, we evaluated the effects of recent preceding dengue infection on risk and severity of subsequent SARS-CoV-2 infection in a population-based cohort of adult Singaporeans, during overlapping dengue and COVID-19 outbreaks driven by concurrent emergence of DENV3 and SARS-CoV-2 Delta/Omicron variants.

## METHODS

### Study Setting and Databases

Singapore is a multiethnic Asian city-state (population, 5.4 million) with significant populations of Chinese, Malay, and Indian ethnicity. Dengue is endemic in tropical Singapore; [10] in 2020, extensive public health interventions introduced to suppress SARS-CoV-2 transmission, including lockdown, coincided with a surge in dengue cases driven predominantly by DENV2 [2, 11] (Supplementary Figure 1). This was followed by a lull in dengue transmission in 2021 [12].

Emergence of the more transmissible SARS-CoV-2 Delta variant resulted in community-wide transmission of COVID-19 in Singapore, which had hitherto been limited to sporadic imported cases and outbreaks in migrant worker dormitories. In January 2022, Omicron BA.1/2 displaced Delta as the predominant strain, resulting in a further surge in SARS-CoV-2 infections from 2022 onwards [13]. Simultaneously, a surge in reported dengue infections in 2022 was attributed to a switch in the predominant serotype from DENV1/2 to DENV3, with close to 90% of sequenced cases on national surveillance attributed to DENV3 [10]. Before the 2022 outbreak, Singapore had not experienced a DENV3 outbreak in the past 3 decades. During the study period of 1 July 2021 through 31 October 2022, overlapping dengue and COVID-19 outbreaks were thus driven by concurrent emergence of DENV3 and SARS-CoV-2 Delta/Omicron variants, to which the majority of the Singaporean population had no prior exposure. The majority of SARS-CoV-2 infections during the study period were vaccine-breakthrough infections, given that ≥90% of the population were previously vaccinated with a 2-dose primary mRNA vaccine series under the national adult vaccination program, with rollout of booster doses in September 2021 during emergence of the Delta wave [14]. Booster vaccination was recommended 6–9 months after completion of the primary vaccine series [14].

National registries for both SARS-CoV-2 and dengue were used to construct cohorts of adult Singaporeans first infected with SARS-CoV-2/dengue during the study period. In Singapore, both dengue and COVID-19 are legally notifiable diseases to the local Ministry of Health (MOH) not later than 24 hours from the time of diagnosis, with corresponding laboratory testing results submitted at the time of notification [10, 15]. Laboratory testing for diagnostic confirmation is widely accessible for both dengue and SARS-CoV-2. During the study period, all Singaporean residents with symptoms of acute respiratory illness were strongly encouraged to seek free confirmatory testing for SARS-CoV-2 and clinical assessment at any health care provider; SARS-CoV-2 testing (polymerase chain reaction [PCR]/rapid-antigen testing) was mandatory for all individuals presenting with acute respiratory illness at any health care provider [15]. Nationally, 91.7% of symptomatic SARS-CoV-2 cases received confirmatory testing within 48 hours of symptom onset [16]. Similarly, diagnostic testing for confirmation of dengue infection is widely available, with NS-1 lateral flow tests widely used as rapid-diagnostic tests to exclude dengue in patients presenting with undifferentiated febrile illness in the primary care setting, and enzyme-linked immunosorbent assay tests for serology and reverse-transcription PCR used in the inpatient setting [2]. Diagnostic laboratories are also required to notify laboratory-confirmed dengue cases (eg, serology/PCR) to MOH within 24 hours of diagnosis [10]. Dengue cases were classified based on acuity of care required (ambulatory vs hospitalization). Date of notification was taken as the date of onset for dengue/SARS-CoV-2 infection.

### Cohort Construction

**Figure 1.**
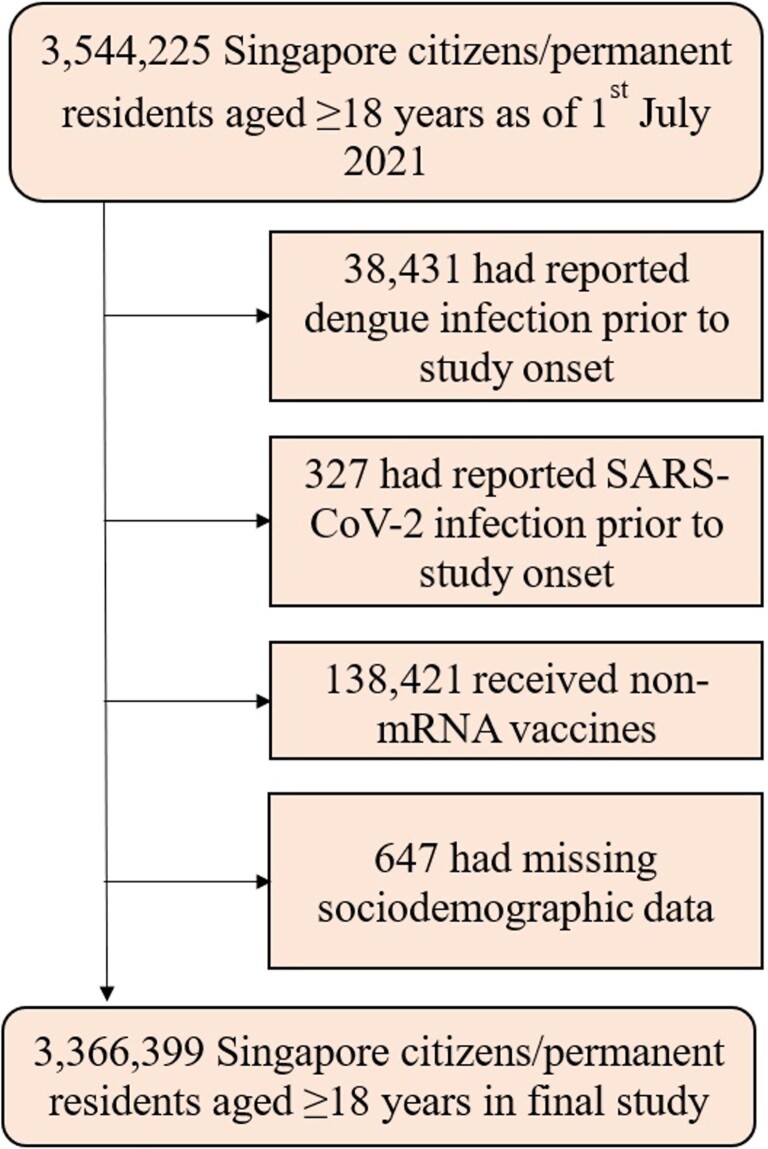
Flowchart of cohort construction.

A flowchart of cohort construction is provided in Figure 1. Singaporean citizens/permanent residents aged ≥18 years at the start of the study period were enrolled. Individuals infected with SARS-CoV-2 before study onset, or with a history of dengue infection in the 5 years before study onset were also excluded to minimize potential confounding introduced by historical infection and subsequent reinfection during the study period. As rates of dengue and COVID-19 coinfection in our local population were extremely low (0.03%), even on systematic testing of all individuals presenting with undifferentiated febrile illness [2, 12], cases of dengue/COVID-19 coinfection were excluded from analysis. Individuals who received non-mRNA COVID-19 vaccinations before the end of the study period were also excluded because they formed a very small nonrepresentative minority of the population (<5%) [14]. Those with missing sociodemographic data were also excluded.

### COVID-19 Outcomes

SARS-CoV-2 infection, COVID-19–related hospitalization, and severe COVID-19 were defined using the national registry of COVID-19 tests and hospitalizations. SARS-COV-2 infection was defined as either a positive PCR or rapid-antigen-test result recorded in the national testing registry; COVID-19–related hospitalization was defined as all-cause hospitalizations occurring within 30 days from a positive COVID-19 result [14]; severe COVID-19 was defined as oxygen supplementation or requiring intensive care unit (ICU) admission. All COVID-19 hospitalizations and severe COVID-19 infections were notified to MOH [15].

### Covariates

The following covariates were included in the main analysis: demographics (age, sex, ethnicity), COVID-19 vaccination status, comorbidities (Charlson Comorbidity Index), immunocompromised status, and socioeconomic status (SES). Comorbidities and immunocompromised status was classified based on ICD-10 codes using the national health care claims database [15]. SES was included given prior studies suggesting that SES impacted both dengue and COVID-19 incidence [3, 17], with housing type used as a surrogate marker of SES [18]. The majority of Singaporeans (≥90%) stay in owner-occupied public housing under a tiered subsidy scheme, with purchase eligibility for more highly subsidized smaller sized flats dependent on monthly household income [18].

### Statistical Analysis

Risks of SARS-CoV-2 infection, COVID-19–related hospitalization and severe COVID-19 based on preceding dengue infection during the study period were estimated using calendar time-scaled Cox regression models, adjusted for demographics (age, sex, ethnicity), SES (housing type), comorbidities, and COVID-19 vaccination status. Person-time was stratified based on onset date of recorded dengue infection; given the varying nature of time elapsed from onset of dengue infection, the same individual could contribute person-time to different time-interval categories. Subgroup analyses by severity of preceding dengue infection (ambulatory vs hospitalized), COVID-19 vaccination status (did not complete primary vaccination series, completed primary vaccination series, completed primary vaccination series, and boosted), and age (18–29 years, 30–59 years, ≥60 years) were also conducted. Vaccination status was defined such that individuals were considered to have completed the primary vaccination series from 7 days after receiving a second dose up to 6 days after receipt of a subsequent booster dose (if applicable). The following sensitivity analyses were performed: (1) restricting the study period to 1 January 2022 onwards, corresponding to predominant transmission of the SARS-CoV-2 Omicron variant; (2) inclusion of prior health care utilization, including number of non–dengue-related hospital admissions and emergency department visits in the preceding year to account for the impact of health care utilization on ascertainment of SARS-CoV-2/dengue infections; and (3) inclusion of area-level SES/population density in the regression model, given prior reported associations between area-level SES/population density and transmission of both dengue [3, 17, 19] and SARS-CoV-2 [17, 20]. Area-level SES was defined as average household income/housing price/average highest education based on postal codes, with prediction performed using a machine-learning algorithm as per previously published methodology [21, 22], using inputs from a representative multiethnic cohort study of adult Singaporeans [23] and historical housing price data for both public housing and private properties [22]. Results for average household income, housing price, and population density were classified by quintiles; for average highest education, results were classified by educational status attained. Population density was defined as average population density within 200 m; the land area of Singapore was subdivided into regular hexagons, each with a circumradius of 200 m and an average area of 0.072 km^2^, and aggregated population size in each hexagon was obtained from the Urban Redevelopment Authority, Singapore [19]. (4) Finally, we conducted a separate supplementary analysis on the population of individuals who had a single reported dengue infection between 1 January 2017 and 31 October 2022, without intervening reinfection. Risks of SARS-CoV-2 infection, COVID-19-related hospitalization, and severe COVID-19 between 1 July 2021 and 31 October 2022 based on preceding dengue infection from 1 January 2017 to 31 October 2022 were estimated using calendar time-scaled Cox regression models; additionally, among those who had a subsequent first SARS-CoV-2 infection between 1 July 2021 and 31 October 2022, a logistic regression model was used to estimate the odds ratio for COVID-19–related hospitalizations and severe COVID-19, stratified based on the time elapsed (<1 year, 1–2 years, ≥3 years) between an individual's first dengue infection and subsequent first SARS-CoV-2 infection. A 95% confidence interval (CI) that excluded a ratio of 1 relative to the reference group was considered evidence of statistical significance. Analyses were conducted using STATA version 18 (Stata Corp., College Station, Texas).

## RESULTS

After applying inclusion and exclusion criteria, a total of 3 366 399 adult Singaporeans (≥18 years) were included in the final study population (Figure 1), contributing a total of 1 399 696 530 person-days of observation over the study period. A total of 13 434 dengue infections were recorded during the study period (Table 1); person-time was stratified into 1 397 860 378 person-days with no prior dengue infection, and 1 836 152 person-days following dengue infection. The majority of dengue cases were nonsevere and did not require hospitalization. Over the same period, a total of 1 253 520 subsequent SARS-CoV-2 infections were recorded, of which 9.2% occurred during Delta-predominant transmission and 90.8% occurred during Omicron-predominant transmission. The mean time elapsed from dengue infection to subsequent SARS-CoV-2 infection was 94.7 days (standard deviation = 33.8). The majority of SARS-CoV-2 infections (96.8%, 1 213 916/1 253 520) were mild and were managed in ambulatory care without hospitalization. The majority of SARS-CoV-2 infections were vaccine-breakthrough infections, with 82.3% (1 032 166/1 253 520) having received a booster vaccine dose prior to infection.

**Table 1.** Characteristics of Individuals With Recorded Dengue/SARS-CoV-2 Infection During Study Period

	Dengue Infection			SARS-CoV-2 Infection		
	No Dengue Infection Recorded During Study Period	Recorded Dengue Infection During Study Period	*P* Value^d^	No SARS-CoV-2 Infection Recorded During Study Period	Recorded SARS-CoV-2 Infection During Study Period	*P* Value^d^
Total, N (%)	3 352 965	13 434		2 112 879	1 253 520	
Gender, N (%)						
Female	1 735 616 (99.6%)	6290 (0.4%)	<.001	1 094 499 (62.8%)	647 407 (37.2%)	.006
Male	1 617 349 (99.6%)	7144 (0.4%)		1 018 380 (62.7%)	606 113 (37.3%)	
Age, N (%)						
18–29 y	549 580 (99.6%)	2312 (0.4%)	<.001	317 442 (57.5%)	234 450 (42.5%)	<.001
30–59 y	1 804 091 (99.6%)	7402 (0.4%)		1 116 768 (61.6%)	694 725 (38.4%)	
≥60 y	999 294 (99.6%)	3720 (0.4%)		678 669 (67.7%)	324 345 (32.3%)	
Ethnicity, N (%)						
Chinese	2 499 077 (99.6%)	10 972 (0.4%)	<.001	1 580 459 (63.0%)	929 590 (37.0%)	<.001
Indian	308 641 (99.7%)	1028 (0.3%)		207 604 (67.0%)	102 065 (33.0%)	
Malay	430 337 (99.8%)	1072 (0.2%)		244 913 (56.8%)	186 496 (43.2%)	
Others^a^	114 910 (99.7%)	362 (0.3%)		79 903 (69.3%)	35 369 (30.7%)	
Socioeconomic status (housing type), N (%)						
Public, 1–3 rooms	880 808 (99.7%)	2453 (0.3%)	<.001	567 816 (64.3%)	315 445 (35.7%)	<.001
Public, 4–5 rooms	1 827 658 (99.7%)	5787 (0.3%)		1 093 055 (59.6%)	740 390 (40.4%)	
Private housing	644 499 (99.2%)	5194 (0.8%)		452 008 (69.6%)	197 685 (30.4%)	
Comorbidity burden, N (%)						
No comorbidities (CCMI = 0)	3 025 317 (99.6%)	11 884 (0.4%)	<.001	1 908 413 (62.8%)	1 128 788 (37.2%)	<.001
Mild comorbidity burden (CCMI 1–3)	294 482 (99.5%)	1358 (0.5%)		183 076 (61.9%)	112 764 (38.1%)	
Moderate-severe comorbidity burden (CCMI ≥4)	33 166 (99.4%)	192 (0.6%)		21 390 (64.1%)	11 968 (35.9%)	
Immunocompromised status^b^						
Nonimmunocompromised	3 264 299 (99.6%)	12 899 (0.4%)	<.001	2 057 260 (62.8%)	1 219 938 (37.2%)	.010
Immunocompromised	88 666 (99.4%)	535 (0.6%)		55 619 (62.4%)	33 582 (37.6%)	
COVID-19 vaccination status (at point of infection)^c^						
Unvaccinated/partially vaccinated	262 748 (99.9%)	168 (0.1%)	<.001	244 890 (93.1%)	18 026 (6.9%)	<.001
Fully vaccinated	260 844 (99.8%)	394 (0.2%)		57 910 (22.2%)	203 328 (77.8%)	
Boosted	2 829 373 (99.5%)	12 872 (0.5%)		1 810 079 (63.7%)	1 032 166 (36.3%)	

Abbreviation: CCMI, Charlson Comorbidity Index.

^a^Includes individuals of other ethnicities or mixed ethnicities.

^b^Immunocompromised status was defined as: presence of solid malignancy, hematologic malignancy, rheumatologic or inflammatory disorders, organ or stem cell transplant, or other intrinsic immune condition or immunodeficiency.

^c^Vaccination status: unvaccinated, partially vaccinated (having received a single dose of mRNA COVID-19 vaccines, either BNT162b2 or mRNA-1273); fully vaccinated (having completed a primary vaccine series of 2 doses of mRNA COVID-19 vaccines); boosted (having received subsequent mRNA vaccine dose(s) after the second dose).

^d^Differences in proportions compared using χ^2^ test.

Risks of SARS-CoV-2 infection, COVID-19–related hospitalization and severe COVID-19 during the study period were estimated in groups with and without prior dengue infection (Table 2). In those without preceding dengue infection, the unadjusted incidence rate of SARS-CoV-2 infection was 894.7 cases per million person-days, whereas among those with preceding dengue infection, the unadjusted incidence rate of SARS-CoV-2 infection was 1537.5 cases per million person-days (Supplementary Table 1). Preceding dengue infection was associated with a modest increase in the risk of subsequent SARS-CoV-2 infection, compared with no preceding dengue infection (aHR = 1.13; 95% CI, 1.08–1.17) (Table 2). Preceding dengue infection was associated with a significantly elevated risk of subsequent COVID-19 hospitalization (aHR = 3.25; 95% CI, 2.78–3.82) and severe COVID-19 (aHR = 3.39; 95% CI, 2.29–5.03**)** (Table 2). Preceding dengue infection was associated with elevated risk of subsequent SARS-CoV-2 infection and COVID-19 hospitalization in those aged 30–59 years and those aged ≥60 years (Table 3, Supplementary Table 2). Preceding dengue infection was associated with elevated risk of subsequent SARS-CoV-2 infection and adverse COVID-19 outcomes across vaccination subgroups (Table 3, Supplementary Table 2). Preceding nonsevere dengue infection was associated with elevated risk of SARS-CoV-2 infection, hospitalization, and severe COVID-19, compared against no prior dengue infection (Table 3). Preceding severe dengue infection was only associated with increased risk of COVID-19 hospitalization, compared against no prior dengue infection (Table 3).

**Table 2.** Risks of SARS-CoV-2 Infection, COVID-19–Related Hospitalization, and Severe COVID-19, With and Without Preceding Dengue Infection

History of Preceding Dengue Infection	Adjusted Hazards Ratio, of SARS-CoV-2 Infection^a^	Adjusted hazards ratio Of COVID-19 Hospitalization^a^	Adjusted hazards ratio of Severe COVID-19^a^
No preceding dengue infection	1.00 (reference)	1.00 (reference)	1.00 (reference)
Preceding dengue infection	1.13 (1.08, 1.17)	3.25 (2.78–3.82)	3.39 (2.29–5.03)

^a^Calendar-time scale Cox regression, controlling for age, gender, ethnicity, socioeconomic status (housing type), comorbidity burden, immunocompromised status, and COVID-19 vaccination status.

**Table 3.** Risks of SARS-CoV-2 Infection, COVID-19–Related Hospitalization, and Severe COVID-19, With and Without Preceding Dengue Infection: in Age, COVID-19 Vaccination, and Dengue Severity Subgroups

History of Preceding Dengue Infection		Adjusted-Hazards-Ratio of SARS-CoV-2 Infection^a^	Adjusted Hazards Ratio, of COVID-19 Hospitalization^a^	Adjusted Hazards Ratio of Severe COVID-19^a^
Age subgroups				
18–29 y	No prior dengue infection	1.00 (reference)	1.00 (reference)	1.00 (reference)
	Prior dengue infection	1.02 (0.94, 1.12)	1.48 (0.37–5.92)	NA^b^
30–59 y	No prior dengue infection	1.00 (reference)	1.00 (reference)	1.00 (reference)
	Prior dengue infection	1.08 (1.03, 1.14)	3.24 (2.20–4.77)	1.40 (0.20–9.98)
≥60 y	No prior dengue infection	1.00 (reference)	1.00 (reference)	1.00 (reference)
	Prior dengue infection	1.31 (1.21, 1.41)	3.33 (2.79–3.97)	3.63 (2.43–5.42)
COVID-19 vaccination subgroups				
Not fully vaccinated	No prior dengue infection	1.00 (reference)	1.00 (reference)	1.00 (reference)
	Prior dengue infection	8.83 (6.33–12.31)	8.52 (5.04–14.41)	4.21 (1.36–13.07)
Fully vaccinated	No prior dengue infection	1.00 (reference)	1.00 (reference)	1.00 (reference)
	Prior dengue infection	1.39 (1.19, 1.64)	3.46 (2.46–4.87)	3.01 (1.43–6.33)
Boosted	No prior dengue infection	1.00 (reference)	1.00 (reference)	1.00 (reference)
	Prior dengue infection	1.08 (1.03–1.12)	2.52 (2.09–3.06)	2.40 (1.44–3.98)
Dengue severity subgroups				
No prior dengue infection		1.00 (reference)	1.00 (reference)	1.00 (reference)
Prior dengue infection	Nonsevere (not requiring hospitalization)	1.14 (1.09–1.18)	3.30 (2.79–3.90)	3.64 (2.43–5.43)
	Severe (requiring hospitalization)	1.00 (0.86–1.16)	2.88 (1.71–4.87)	1.30 (0.18–9.25)

^a^Calendar-time scale Cox regression, controlling for age, gender, ethnicity, socioeconomic status (housing type), comorbidity burden, immunocompromised status, and COVID-19 vaccination status.

^b^Not computed because of an absence of cases.

In sensitivity analyses restricting the study duration to the period of SARS-CoV-2 Omicron transmission, increased risk of SARS-CoV-2 infection, COVID-19 hospitalization, and severe COVID-19 was still observed with preceding dengue infection (Supplementary Table 3). Inclusion of prior non–dengue-related health care utilization and area-level SES/population density produced hazard ratios that replicated primary estimates (Supplementary Table 4). When the cohort was expanded to incorporate all individuals who had a single reported dengue infection between 1 January 2017 and 31 October 2022, without intervening reinfection, higher risk of SARS-CoV-2 infection (aHR = 1.10; 95% CI, 1.08–1.11), COVID-19–related hospitalization (aHR = 1.50; 95% CI, 1.39–1.62), and severe COVID-19 (aHR = 1.64; 95% CI, 1.38–1.96) was still observed among those with preceding dengue infection, compared to those without preceding dengue infection (Supplementary Table 5). Decreased odds of COVID-19 hospitalization and severe COVID-19 following subsequent SARS-CoV-2 infection were observed with longer time elapsed from prior dengue infection, with lower risk of COVID-19 hospitalization observed among those infected with dengue 1–2 years (aOR = 0.39; 95% CI, .32–.48) and ≥3 years prior (aOR = 0.33; 95% CI, .27–.41), compared with those infected with dengue <1 year prior (Supplementary Table 6).

## DISCUSSION

In a national population-based cohort of adult Singaporeans, during overlapping dengue and COVID-19 outbreaks driven by concurrent emergence of DENV3 and SARS-CoV-2 Delta/Omicron variants, increased risk of SARS-CoV-2 infection, COVID-19 hospitalization, and severe COVID-19 was observed following recent preceding dengue infection. Previously, serological proof of prior dengue infection was associated with twice the risk of clinically apparent COVID-19 in a cohort recruited from Amazonian Brazil, during transmission of ancestral SARS-CoV-2 variants and predating the availability of COVID-19 vaccines [3]. Our results suggest that recent preceding dengue infection remains associated with increased risk of SARS-CoV-2 infection and adverse COVID-19 outcomes, even in the context of milder Omicron infection [24] and the protective effect of vaccination and boosting [14]. To the best of our knowledge, no other studies have evaluated the impact of preceding dengue infection on severity of COVID-19 in the Delta/Omicron era. Increased risk of symptomatic dengue following prior COVID-19 was observed in a small cohort study from North India, predominantly comprised of healthcare workers [25]. Additionally, the burden of health loss following COVID-19 is highest in the postacute phase of infection because of persistence of long-term sequelae (“long COVID-19”) [26]. Given that the greatest burden of postacute sequelae accrues in individuals hospitalized for COVID-19 [27–29], increased risk of COVID-19 hospitalization following prior dengue infection may increase subsequent morbidity accruing from long COVID-19 in countries where dengue is endemic. Although prior history of endemic tropical infections, including dengue, was not associated with long-term symptoms following COVID-19 at 12-month follow-up [30], the relatively young age (mean age, 40 years) of the cohort may have been a protective factor, given that older adults are at higher risk of postacute sequelae following COVID-19 [26–29]. Our results suggesting increased risk of adverse COVID-19 outcomes following prior dengue infection are of public health significance, especially in less well-resourced health care systems that may struggle with the burden of health care utilization arising from acute and postacute sequelae following overlapping dengue and COVID-19 outbreaks [1, 31, 32].

Increased risk of adverse COVID-19 outcomes following dengue infection may potentially be attributable to overlapping sociodemographic factors predisposing at-risk individuals to both dengue infection and severe COVID-19, or may suggest the possibility of increased risk of subsequent severe COVID-19 through an antibody dependent-like enhancement mechanism, after a preceding infection. Age and SES affect dengue and COVID-19 incidence and can potentially lead to spatiotemporal overlap [3, 19]; however, increased risk of adverse COVID-19 outcomes following dengue infection persisted even after adjustment for age and SES in our analyses. Serological cross-reactivity between dengue and SARS-CoV-2 has been extensively reported in the literature, though estimates of the extent of cross-reactivity differ substantially. Although minimal cross-reactivity between SARS-CoV-2 and dengue has been reported in some studies [5], other studies report false positivity rates of 5%–22% for SARS-CoV-2 in individuals with dengue infection [6–8]. Anti-DENV antibodies can potentially bind to the SARS-CoV-2 spike protein [7]; however, whether such cross-reactivity can result in cross-protection or rather, increased severity via antibody-dependent enhancement, is currently unclear. The phenomenon of antibody-dependent enhancement is attributable to the presence of nonneutralizing antibodies or antibodies at subneutralizing levels; deposition of antigen-antibody complexes in tissue precipitates chronic inflammation and predisposes to increased disease severity [33]. In vitro data suggested that anti-DENV antibodies retain the ability to bind to the SARS-CoV-2 spike receptor–binding domain, but this was insufficient to achieve cross-protection via neutralization of SARS-CoV-2 [8]. Although we describe an association between prior dengue infection and subsequent adverse COVID-19 outcomes, our data do not show direct causal association or prove irrefutably an immunological mechanism, a hypothesis that requires validation in future prospective cohorts.

The present study has several strengths. Comprehensive nationwide registries were used to classify SARS-CoV-2 and dengue infection status, with supporting diagnostic confirmation of infection, potentially reducing misclassification bias. A large number of potential confounders were controlled for, including sociodemographic factors, comorbidities, health care utilization, and area-level SES/population density. However, several limitations remain. Our analysis was restricted to the period corresponding to emergence of DENV3 in Singapore; as such, results may not be generalizable to infections caused by other dengue serotypes. SARS-CoV-2 variant and dengue serotype was imputed based on period of predominant transmission and not individual-level sequencing, resulting in potential misclassification. Our findings that preceding dengue infection was associated with elevated risk of subsequent SARS-CoV-2 infection and adverse COVID-19 outcomes across vaccination subgroups cannot be generalized to individuals who received non-mRNA vaccines. Results of serological testing were not available; hence, we did not have information regarding level of preexisting anti-DENV antibodies in individual cases, or its temporality. Only symptomatic dengue and SARS-CoV-2 infections would have been tested by health care providers and reported to our local MOH; however, a proportion of infections may be asymptomatic and hence unrecognized or underreported [34]. Additionally, criteria for COVID-19 hospitalization were progressively relaxed during the Omicron period (versus Delta) because of the large number of infections and relatively milder disease. However, in sensitivity analyses restricting the study period to the period of Omicron transmission, increased risk of SARS-CoV-2 infection and adverse COVID-19 outcomes was still observed with preceding dengue infection.

## CONCLUSION

Increased risk of SARS-CoV-2 infection, COVID-19 hospitalization and severe COVID-19 was observed following preceding dengue infection in a national population-based cohort of adult Singaporeans during overlapping dengue and COVID-19 outbreaks driven by concurrent emergence of DENV3 and SARS-CoV-2 Delta/Omicron variants. This is of significance particularly in tropical countries where seasonal dengue outbreaks can overlap with SARS-CoV-2 transmission.

## Supplementary Material

ofae397_Supplementary_Data

## Supplementary Data

Supplementary materials are available at *Open Forum Infectious Diseases* online. Consisting of data provided by the authors to benefit the reader, the posted materials are not copyedited and are the sole responsibility of the authors, so questions or comments should be addressed to the corresponding author.

## Notes

***Author contributions.*** L. E. W. contributed to literature search and writing of the manuscript. N. T., J. T. L., B. D., C. C., L. C. N., P. Y. C., Y. S. L., D. C. L., and K. B. T. contributed to critical review and editing of the manuscript. D. C. L. and K. B. T. provided supervision. K. B. T., L. E. W., and N. T. contributed to study design. N. T. and B. D. contributed to data collection, and N. T. contributed to data analysis. All authors had full access to all the data in the study and take responsibility for the decision to submit for publication. L. E. W. and N. T. directly accessed and verified the underlying data reported in the manuscript. The corresponding author (L. E. W.) is the guarantor and accepts full responsibility for the work and/or the conduct of the study, had access to the data, and controlled the decision to publish. The corresponding author (L. E. W.) attests that all listed authors meet authorship criteria and that no others meeting the criteria have been omitted.

***Data availability.*** The databases with individual-level information used for this study are not publicly available because of personal data protection. Deidentified data can be made available for research, subject to approval by the Ministry of Health of Singapore. All inquiries should be sent to the corresponding author.

***Patient Consent Statement.*** This study was done as part of national public health research under the Infectious Diseases Act, Singapore; ethics review by an institutional review board was not required.

***Financial support*.** This work was not grant-funded. L. E. W. is supported by the Singhealth Regional Health Systems PULSES Fellowship. J. T. L. is supported by the Ministry of Education (MOE), Singapore Start-up Grant.
