# Supplementary material for: Effects of Recent Prior Dengue Infection on Risk and Severity of Subsequent SARS-CoV-2 Infection: A Retrospective Cohort Study
Source: Open Forum Infect Dis. 2024 Jul 13;11(8):ofae397. doi: 10.1093/ofid/ofae397 (PMC11293429; doi:10.1093/ofid/ofae397)
Supplement: ofae397_Supplementary_Data [file ofae397_supplementary_data.zip › dengue covid supp tables r1.2.docx]

# Supplementary Table 1: Unadjusted incidence of SARS-CoV-2 infection, COVID-19-related hospitalization and severe COVID-19 during study period and Omicron-predominant transmission, with and without preceding dengue infection

| **History of preceding dengue infection** | **Person-days** | **Number of reported SARS-CoV-2 infections** | **Unadjusted incidence rate of reported SARS-CoV-2 infection (per million person-days)** | **Number of reported COVID-19 hospitalizations** | **Unadjusted incidence rate of COVID-19 hospitalizations (per million person-days)** | **Number of severe-COVID-19** | **Unadjusted incidence rate of severe COVID-19 (per million person-days)** |
| --- | --- | --- | --- | --- | --- | --- | --- |
| **Entire study period (1st Jul 2021- 31st Oct 2022)** | | | | | | | |
| No preceding dengue infection | 1,397,860,378 | 1,250,697 | 894.7 | 39,371 | 28.2 | 7,843 | 5.6 |
| Preceding dengue infection | 1,836,152 | 2,823 | 1537.5 | 233 | 126.9 | 39 | 21.2 |
| **Omicron-predominant transmission (1st Jan to 31st Oct 2022)** | | | | | | | |
| No preceding dengue infection | 787,472,713 | 1,134,984 | 1,441.3 | 26,437 | 33.6 | 4,916 | 6.2 |
| Preceding dengue infection | 1,735,656 | 2,766 | 1,593.6 | 204 | 117.5 | 30 | 17.3 |

# Supplementary Table 2: Unadjusted incidence of SARS-CoV-2 infection, COVID-19-related hospitalization and severe COVID-19, with and without preceding dengue infection: in age, COVID-19 vaccination, and dengue severity subgroups

| **History of preceding dengue infection** | | **Unadjusted incidence rate of reported SARS-CoV-2 infection (per million person-days)** | **Unadjusted incidence rate of COVID-19 hospitalizations (per million person-days)** | **Unadjusted incidence rate of severe COVID-19 (per million person-days)** |
| --- | --- | --- | --- | --- |
| **Age subgroups** | |  |  |  |
| 18-29y | No prior dengue infection | 1,035.6 | 6.3 | 0.2 |
|  | Prior dengue infection | 1,637.8 | 6.3 | 0.0 |
| 30-59y | No prior dengue infection | 921.4 | 11.1 | 1.3 |
|  | Prior dengue infection | 1,526.7 | 37.5 | 2.0 |
| ≥60y | No prior dengue infection | 771.1 | 70.6 | 16.3 |
|  | Prior dengue infection | 1,496.8 | 379.1 | 72.7 |
| **COVID-19 vaccination subgroups** | |  |  |  |
| Not fully vaccinated | No prior dengue infection | 97.9 | 31.3 | 12.1 |
|  | Prior dengue infection | 2,081.0 | 1207.0 | 291.3 |
| Fully vaccinated | No prior dengue infection | 414.2 | 28.5 | 5.2 |
|  | Prior dengue infection | 1,232.1 | 320.5 | 71.2 |
| Boosted | No prior dengue infection | 1,422.7 | 27.1 | 4.3 |
|  | Prior dengue infection | 1,555.3 | 95.1 | 13.2 |
| **Dengue severity subgroups** | |  |  |  |
| No prior dengue infection | | 894.7 | 28.2 | 5.6 |
| Prior dengue infection | Non-severe (not requiring hospitalization) | 1,552.0 | 125.1 | 21.8 |
|  | Severe (requiring hospitalization) | 1,362.5 | 149.0 | 14.2 |

# Supplementary Table 3: Risks of SARS-**Co**V-2 infection, COVID-19-related hospitalization and severe COVID-19, with and without preceding dengue infection, during Omicron-predominant transmission

| **History of preceding dengue infection** | **Adjusted-hazards-ratio, aHR, of SARS-CoV-2 infection^a^** | **Adjusted-hazards-ratio, aHR, of COVID-19 hospitalization^a^** | **Adjusted-hazards-ratio, aHR, of severe COVID-19^a^** |
| --- | --- | --- | --- |
| No preceding dengue infection | 1.00 (reference) | 1.00 (reference) | 1.00 (reference) |
| Preceding dengue infection | 1.11 (1.06, 1.15) | 2.81 (2.38, 3.33) | 2.38 (1.52, 3.74) |

^a^ Calendar-time scale cox regression, controlling for age, gender, ethnicity, socioeconomic status (housing type), comorbidity burden, immunocompromised status, and COVID-19 vaccination status

# Supplementary Table 4: Risks of SARS-CoV-2 infection, COVID-19-related hospitalization and severe COVID-19, with and without preceding dengue infection; incorporating healthcare utilization, area-level socioeconomic status and population density

| **History of preceding dengue infection** | **Person-days** | **Number of reported SARS-CoV-2 infections** | **Unadjusted incidence rate of reported SARS-CoV-2 infection (per million person-days)** | **Adjusted-hazards-ratio, aHR, of SARS-CoV-2 infection** | **Number of reported COVID-19 hospitalizations** | **Unadjusted incidence rate of COVID-19 hospitalizations (per million person-days)** | **Adjusted-hazards-ratio, aHR, of COVID-19 hospitalization** | **Number of severe-COVID-19** | **Unadjusted incidence rate of severe COVID-19 (per million person-days)** | **Adjusted-hazards-ratio, aHR, of severe COVID-19** |
| --- | --- | --- | --- | --- | --- | --- | --- | --- | --- | --- |
| **Model 1 (incorporating healthcare utilisation)^a^** | | | | | | | | | | |
| No preceding dengue infection | 1,397,860,378 | 1,250,697 | 894.7 | 1.00 (reference) | 39,371 | 28.2 | 1.00 (reference) | 7,843 | 5.6 | 1.00 (reference) |
| Preceding dengue infection | 1,836,152 | 2,823 | 1537.5 | 1.10 (1.06, 1.14) | 233 | 126.9 | 1.79 (1.53, 2.10) | 39 | 21.2 | 1.58 (1.06, 2.34) |
| **Model 2 (incorporating area-level socioeconomic status and population density)^a^** | | | | | | | | | | |
| No preceding dengue infection | 1,201,083,226 | 1,131,892 | 942.4 | 1.00 (reference) | 35,009 | 29.1 | 1.00 (reference) | 6,939 | 5.8 | 1.00 (reference) |
| Preceding dengue infection | 1,376,422 | 2,188 | 1589.6 | 1.09 (1.05, 1.14) | 202 | 146.8 | 3.31 (2.78, 3.93) | 35 | 25.4 | 3.47 (2.28, 5.28) |
| **Model 3 (incorporating healthcare utilisation, area-level socioeconomic status and population density)^a^** | | | | | | | | | | |
| No preceding dengue infection | 1,201,083,226 | 1,131,892 | 942.4 | 1.00 (reference) | 35,009 | 29.1 | 1.00 (reference) | 6,939 | 5.8 | 1.00 (reference) |
| Preceding dengue infection | 1,376,422 | 2,188 | 1589.6 | 1.06 (1.02, 1.11) | 202 | 146.8 | 1.78 (1.50, 2.11) | 35 | 25.4 | 1.59 (1.04, 2.42) |

^a^ Model 1: Calendar-time scale cox regression, controlling for age, gender, ethnicity, socioeconomic status (housing type), comorbidity burden, immunocompromised status, COVID-19 vaccination status, and individual healthcare utilization (number of non-dengue-related hospital admissions and emergency-department visits in the preceding year).

Model 2: Calendar-time scale cox regression, controlling for age, gender, ethnicity, socioeconomic status (housing type), comorbidity burden, immunocompromised status, COVID-19 vaccination status, area-level socioeconomic status (average household income/housing price/highest education) and population density. Excludes individuals missing area-level data

Model 3: Calendar-time scale cox regression, controlling for age, gender, ethnicity, socioeconomic status (housing type), comorbidity burden, immunocompromised status, COVID-19 vaccination status, individual healthcare utilization (number of non-dengue-related hospital admissions and emergency-department visits in the preceding year), area-level socioeconomic status (average household income/housing price/highest education) and population density. Excludes individuals missing area-level data

# Supplementary Table 5: Risks of SARS-CoV-2 infection, COVID-19-related hospitalization and severe COVID-19, with and without preceding dengue infection, amongst individuals with a single reported dengue infection between 1 January 2017 to 31 October 2022

| **History of preceding dengue infection** | **Number of reported SARS-CoV-2 infections** | **Unadjusted incidence rate of reported SARS-CoV-2 infection (per million person-days)** | **Adjusted-hazards-ratio, aHR, of SARS-CoV-2 infection^a^** | **Number of reported COVID-19 hospitalizations** | **Adjusted-hazards-ratio, aHR, of COVID-19 hospitalization^a^** | **Number of severe-COVID-19 cases** | **Adjusted-hazards-ratio, aHR, of severe COVID-19^a^** |
| --- | --- | --- | --- | --- | --- | --- | --- |
| No preceding dengue infection | 1,250,688 | 894.7 | 1.00 (reference) | 39,371 | 1.00 (reference) | 7,843 | 1.00 (reference) |
| Preceding dengue infection | 17,259 | 1049.4 | 1.10 (1.08, 1.11) | 716 | 1.50 (1.39, 1.62) | 139 | 1.64 (1.38, 1.96) |

^a^ Calendar-time scale cox regression, controlling for age, gender, ethnicity, socioeconomic status (housing type), comorbidity burden, immunocompromised status, and COVID-19 vaccination status

# Supplementary Table 6: Risks of COVID-19-related hospitalization and severe COVID-19, stratified by time elapsed between SARS-CoV-2 infection and prior dengue, in individuals with a single reported dengue infection (1 Jan 2017 – 31 Oct 2022) followed by SARS-CoV-2 infection between 1 Jul 2021 – 31 Oct 2022 (N=17,259)

| **Time elapsed since preceding dengue infection** | **Number of cases (single reported dengue infection 2017-2022, followed by SARS-CoV-2 infection)** | **Number of reported COVID-19 hospitalizations, N(%)** | **Adjusted-odds-ratio, aOR (95%CI), of COVID-19 hospitalization^a^** | **Number of severe-COVID-19 cases, N(%)** | **Adjusted-odds-ratio, aOR (95%CI), of severe COVID-19^a^** |
| --- | --- | --- | --- | --- | --- |
| Dengue infection <1 year prior to SARS-CoV-2 infection | 3,118 | 261 | 1.00 (reference) | 46 | 1.00 (reference) |
| Dengue infection 1-2 years prior to SARS-CoV-2 infection | 6,326 | 245 | 0.39 (0.32, 0.48) | 48 | 0.58 (0.38, 0.90) |
| Dengue infection ≥ 3 years prior to SARS-CoV-2 infection | 7,815 | 210 | 0.33 (0.27, 0.41) | 45 | 0.60 (0.39, 0.95) |

^a^ Logistic regression, controlling for age, gender, ethnicity, socioeconomic status (housing type), comorbidity burden, immunocompromised status, COVID-19 vaccination status at point of SARS-CoV-2 infection
