# Supplementary figures and images for: Effects of Recent Prior Dengue Infection on Risk and Severity of Subsequent SARS-CoV-2 Infection: A Retrospective Cohort Study
Source: Open Forum Infect Dis. 2024 Jul 13;11(8):ofae397. doi: 10.1093/ofid/ofae397 (PMC11293429; doi:10.1093/ofid/ofae397)

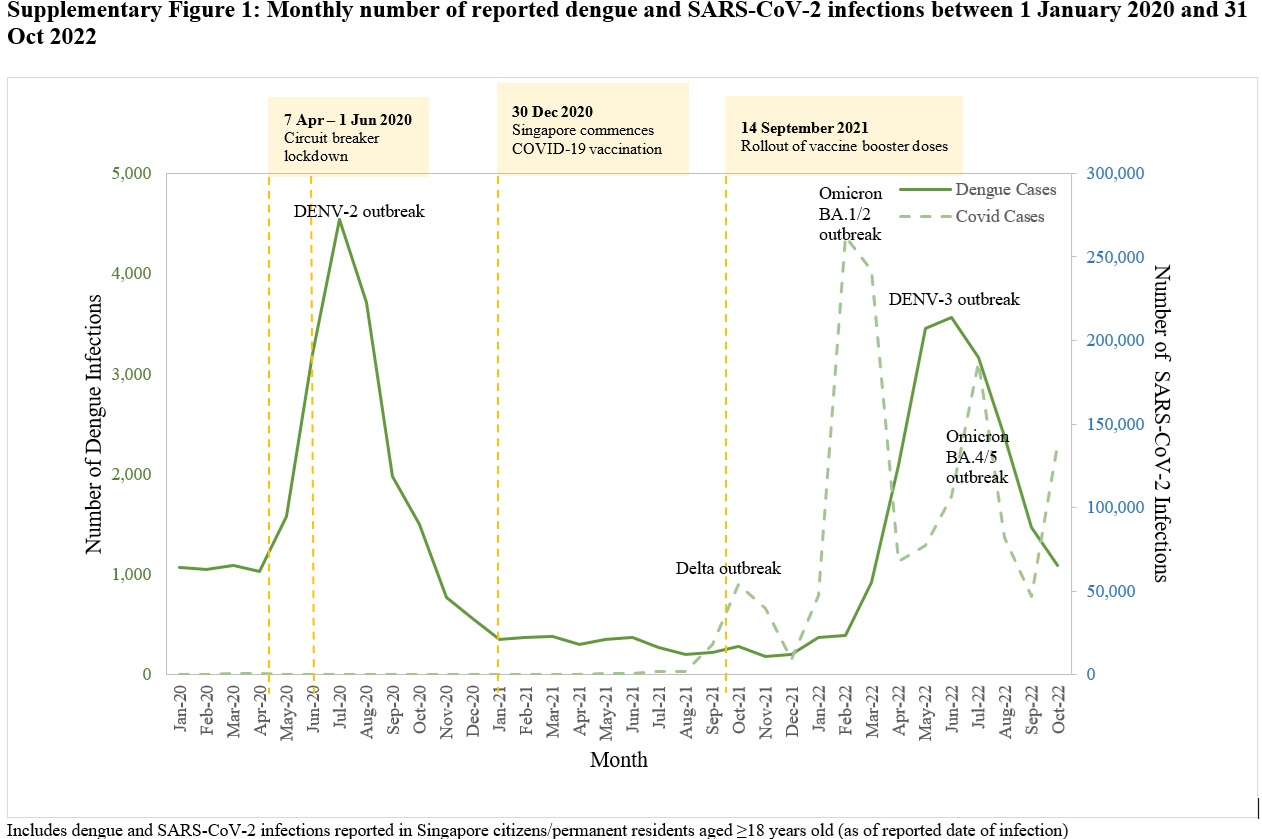

Supplement: ofae397_Supplementary_Data [file ofae397_supplementary_data.zip › Supp Fig 1.jpg]
